# Reasons for Acceptance or Rejection of Online Record Access Among Patients Affected by a Severe Mental Illness: Mixed Methods Study

**DOI:** 10.2196/51126

**Published:** 2024-02-05

**Authors:** Julian Schwarz, Eva Meier-Diedrich, Katharina Neumann, Martin Heinze, Yvonne Eisenmann, Samuel Thoma

**Affiliations:** 1 Department of Psychiatry and Psychotherapy Center for Mental Health, Immanuel Hospital Rüdersdorf Brandenburg Medical School Theodor Fontane Rüdersdorf Germany; 2 Center for Health Service Research Brandenburg Brandenburg Medical School Theodor Fontane Rüdersdorf Germany; 3 Faculty of Health Sciences Brandenburg Brandenburg Medical School Theodor Fontane Neuruppin Germany

**Keywords:** open notes, patient-clinician relations, electronic health record, clinical notes, visit notes, patient participation, online record access, mental illness, patient portal, mental health, qualitative interview, patient education

## Abstract

**Background:**

Over the past few years, online record access (ORA) has been established through secure patient portals in various countries, allowing patients to access their health data, including clinical notes (“open notes”). Previous research indicates that ORA in mental health, particularly among patients with severe mental illness (SMI), has been rarely offered. Little is known about the expectations and motivations of patients with SMI when reading what their clinicians share via ORA.

**Objective:**

The aim of this study is to explore the reasons why patients with SMI consider or reject ORA and whether sociodemographic characteristics may influence patient decisions.

**Methods:**

ORA was offered to randomly selected patients at 3 university outpatient clinics in Brandenburg, Germany, which exclusively treat patients with SMI. Within the framework of a mixed methods evaluation, qualitative interviews were conducted with patients who chose to participate in ORA and those who declined, aiming to explore the underlying reasons for their decisions. The interviews were transcribed and analyzed using thematic analysis. Sociodemographic characteristics of patients were examined using descriptive statistics to identify predictors of acceptance or rejection of ORA.

**Results:**

Out of 103 included patients, 58% (n=60) wished to read their clinical notes. The reasons varied, ranging from a desire to engage more actively in their treatment to critically monitoring it and using the accessible data for third-party purposes. Conversely, 42% (n=43) chose not to use ORA, voicing concerns about possibly harming the trustful relationship with their clinicians as well as potential personal distress or uncertainty arising from reading the notes. Practical barriers such as a lack of digital literacy or suspected difficult-to-understand medical language were also named as contributing factors. Correlation analysis revealed that the majority of patients with depressive disorder desired to read the clinical notes (*P*<.001), while individuals with psychotic disorders showed a higher tendency to decline ORA (*P*<.05). No significant group differences were observed for other patient groups or characteristics.

**Conclusions:**

The adoption of ORA is influenced by a wide range of motivational factors, while patients also present a similar variety of reasons for declining its use. The results emphasize the urgent need for knowledge and patient education regarding factors that may hinder the decision to use ORA, including its practical usage, its application possibilities, and concerns related to data privacy. Further research is needed to explore approaches for adequately preparing individuals with SMI to transition from their inherent interest to active engagement with ORA.

**Trial Registration:**

German Clinical Trial Register DRKS00030188; https://drks.de/search/en/trial/DRKS00030188

## Introduction

In recent years, several countries have established secure patient portals to enable online record access (ORA), allowing patients to view their health data, including clinical notes (“open notes”) from their health care providers [1]. The United States, Canada, and Scandinavian countries, particularly Estonia, Sweden, and Norway, have been at the forefront, providing access to a significant number of patients across multiple regions [2,3]. More recently, the United Kingdom introduced the NHS app, offering access to primary care provider medical records since just last year [4]. In Germany, offering ORA was made mandatory for statutory health insurance providers by 2021, although the inclusion of open notes remains uncertain [5,6].

Research conducted in general health settings indicates clear benefits of patient access to clinical notes, including improved treatment satisfaction, transparency, patient engagement, patient-clinician communication, and health literacy [7-9]. Additionally, ORA enhances medication adherence and security in patients, helps patients to identify and correct treatment errors [10], increases a sense of control over the treatment, and reduces anxieties regarding the treatment process [11]. Health care providers generally view ORA as a valuable tool to promote patient engagement, even though it may be connected to an additional workload related to documentation and communication [12].

Studies conducted in mental health generally yield similar results to those in general health settings, but they also highlight distinct ethical and practical challenges [13,14]. For example, these challenges encompass navigating disagreements between patients and health care professionals (HCPs) regarding clinical notes, as well as discussing exceptions to or limitations of access for patient groups with specific diagnoses or acute conditions, such as severe mental illnesses (SMI) [15,16]. SMI is commonly characterized by conditions including (1) nonorganic psychoses, bipolar disorders, personality disorders, or severe chronic depression; (2) a prolonged psychiatric history involving multiple hospitalizations or outpatient treatments; or (3) moderate impairment in work and leisure activities alongside mild impairment in basic needs [17]. Clinicians often hold reservations about offering ORA to patients affected by SMI. Their concerns primarily revolve around the apprehension that ORA might contribute to self-harming or violent behaviors, especially in patients with whom establishing a trusting relationship is challenging [14]. Patient surveys, however, have demonstrated that individuals with SMI using ORA also exhibit an improved understanding of medication prescriptions, higher medication adherence, and a greater sense of control over their medication [18]. Nevertheless, the adoption of ORA among patients with SMI remains lower compared to those receiving treatment for somatic conditions [18,19].

Apart from assumed positive effects on the patient-clinician relationship and concerns regarding data security, little is known about the motivations and expectations of patients with SMI toward ORA [14,20]. Examining these aspects is crucial for addressing the barriers that hinder the widespread acceptance of ORA among patients with SMI. Therefore, this study aims to thoroughly explore the reasons why patients with SMI consider or reject ORA and whether sociodemographic characteristics may influence patient decisions. More specifically, the following research questions will be addressed (1) which factors influence the decision of patients with SMI, who are offered access to their clinical notes, to either embrace or reject this option? (2) Does the decision for or against ORA in the context of SMI relate to any patient characteristics?

## Methods

### Design

This study is part of the PEPPPSY project (Piloting and Evaluation of a Participatory Patient-Accessible Electronic Health Record in Psychiatry and Somatics; 2021-2023) that focuses on piloting and evaluating a participatory patient record in psychiatry and somatic medicine [21,22]. It aims to examine the development, implementation, processes, and outcomes of the corresponding patient portal, also known as PEPPPSY, from the perspectives of patients and HCPs. Based on a concurrent mixed methods design, a qualitative methodology was used to comprehensively explore the reasons for and against ORA as well as the potential benefits and challenges [23]. This was complemented by a quantitative analysis to study the possible association between the decision for or against ORA and patient characteristics. Qualitative and quantitative data were analyzed separately. The concurrent design was chosen in order to obtain a comprehensive view of the research question [23], as it simultaneously provides in-depth, qualitative insights into the reasons for acceptance or rejection of ORA and broad, quantitatively measurable data on the patient characteristics in relation to this approval or rejection.

### Ethical Considerations

The ethics approval from the Ethics Committee of the Brandenburg Medical School (MHB) was obtained (E-01-20210727), and the study was registered with the German Clinical Trial Register (DRKS00030188).

### PEPPPSY App

The patient portal PEPPPSY was developed as part of a research collaboration between the Norwegian University of Science and Technology (NTNU) and the MHB. It emerged from an iterative process of participatory design, development, application, and evaluation [21,22]. PEPPPSY’s primary function is to provide patients with secure, 2-factor authenticated access to their physician’s notes, but it also includes other information such as laboratory results and a list of prescribed medications. In the current second phase of the pilot, PEPPPSY is being expanded to serve a broader patient population, offering additional features such as a messaging function to allow communication between patients and clinicians concerning the open notes.

### Study Setting

The study was conducted at 3 psychiatric outpatient university clinics of psychiatry and psychotherapy at MHB, Immanuel Klinik Rüdersdorf. Located in the metropolitan region Berlin/Brandenburg, the clinics are responsible for providing mental health care services to approximately 255,000 inhabitants in the catchment area. These outpatient clinics offer specialized care for patients who require a comprehensive, multidisciplinary approach due to the nature, severity, or duration of their conditions. The eligibility for receiving treatment in these psychiatric outpatient clinics is based on specific diagnoses, including SMI and other diagnoses, as determined by the insurance providers and the hospital association.

### Recruitment and Sampling

From January to June 2023, eligible patients were randomly selected from the 3 study centers by the PEPPPSY research team consecutively. Participants had to meet the following inclusion criteria: age 18 years or older, diagnosed with SMI and confirmed by an external report, and currently receiving treatment in an outpatient psychiatric clinic. Exclusion criteria were previous ORA use related to mental health issues; acute psychiatric conditions or symptoms such as disorientation, severe delusions, hallucinations, katatonia, or agitation that may be associated with significant impairment of cognitive and social functioning; or acute self-harm or harm to others. Eligible patients were informed about the study and written informed consent was obtained. The former included detailed information about ORA, such as how it works, the health information it provides, and offers to participate in (1) this qualitative study and (2) the intervention part of the study, that is, to try out ORA for a period of several months in the study setting described.

### Data Collection

The interviews that form the basis of the data in this study were conducted immediately after informed consent was obtained, which included a decision about whether or not participants wished to receive ORA. Sociodemographic data were collected, followed by face-to-face interviews. Information on the diagnoses of the study participants according to the International Classification of Diseases (ICD) was taken from the patient’s medical records. The interviews were performed at the aforesaid outpatient clinics by the authors and psychiatrists, ST and JS, who were not the outpatient treatment providers of the participating patients. The interviewers conducted the interviews out of genuine interest in understanding why ORA is accepted or rejected. For the interviews, a semistructured approach based on an interview guide was chosen in order to ensure the comparability of the interviews. This interview guide (see Multimedia Appendix 1) was developed deductively with the participation of all researchers on the basis of a desktop study (or literary research) on the topic of acceptance of ORA among patients with psychiatric disorders using Google Scholar and PubMed [24]. The interview guide was then tested in 2 pilot sessions with patients within the authors’ institution. Since no changes to the guide were necessary, the sample interviews could be included in the analysis.

The interviews explored each patient’s reasons for acceptance or rejection of ORA usage and had a mean duration of 11.3 (SD 4.5) minutes. In addition to the interviews, the researchers also took field notes, which were later included in the data analysis. Data collection continued until thematic saturation of categories was reached, which occurred when no new themes emerged from the transcripts. The saturation of categories was defined as the point at which no new codes appeared and the meaning of the category and subcategories were established [25].

### Data Analysis

The interviews were transcribed verbatim and pseudonymized (JS and ST) and analyzed with thematic analysis (JS, ST, and KN) using the MAXQDA Software (Verbi Software Ltd). Thematic analysis is a flexible approach that was used to inductively (“bottom up”) analyze data gathered from semistructured interviews [23]. Data analysis was initially conducted by 2 researchers on each interview individually and verified for consensus; a third person joined in when coders could not reach consensus. The analysis proceeded in six steps (1) familiarizing oneself or becoming familiar with the data, (2) generating initial codes, (3) generating initial themes, (4) reviewing themes, (5) defining and adequately naming themes related to the research questions, and (6) formulating key concepts. After all the themes were generated for each of the interviews, they were divided into 2 groups, reasons for acceptance or rejection of ORA. Subsequently, these themes were clustered within these groups and overarching categories and subcategories were formed. This was a recursive process where different categories were repeatedly tested for coherence and differentiability from the other categories and subcategories. In the final step, 2 researchers jointly selected the most relevant and succinct quotes from the subjects for each of the categories and subcategories. For the group of acceptance of ORA, 4 categories and 13 subcategories were formed. For the group of rejection of ORA, 5 categories and 13 subcategories were formed. For quality assurance purposes, the Consolidated Criteria for Reporting Qualitative research (COREQ) checklist was used (see Multimedia Appendix 2) [26].

The sociodemographic data of participants were analyzed according to their group affiliation (acceptance vs rejection ORA). Descriptive statistics were used to examine possible differences in sociodemographic characteristics between groups using R software (R Core Team) [27], which is available license-free. These between-group differences and their significance were assessed using chi-square test and *t* test [28]. No data were excluded from the data analysis.

## Results

### Sociodemographic Data

Out of the 124 eligible patients, 83.1% (n=103) agreed to participate in the study about reasons to use or not to use ORA. Sociodemographics are summarized in Table 1. The respondents had an average age of 46.1 (ranging from 19 to 86) years. The majority of the participants were women (n=64, 62%) with an average age of 47.2 years. Respondents who were men had an average age of 44.2 years. Among the approached patients, 58% (n=60) expressed a desire to use ORA, while 42% (n=43) declined ORA. When differentiating for gender, 56% (n=36) of the respondents who were women agreed to participate, while 44% (n=28) declined. Among respondents who were men, 62% (n=24) agreed to participate, while 38% (n=15) declined. The willingness to participate was highest among younger respondents (aged 18 to 39 years) and among patients aged 50 to 59 years. In terms of diagnosis, a high willingness to participate was observed among individuals with affective disorders (ICD 10, F3 [mood (affective) disorders]) at 91% (n=48; *P*<.001). The lowest agreement was found among individuals with schizophrenia, schizotypal, and delusional disorders (ICD 10, F2 [schizophrenia, schizotypal, and delusional disorders]) at 35% (n=6; *P*=.01).

**Table 1:** Sociodemographic characteristics of the study sample (N=103).

Characteristics			All (N=103)		Do you want online record access?				*P* value	
					Yes (n=60)		No (n=43)			
**Gender, n (%)**										.68
	Women	64 (62.1)		36 (56.2)		28 (43.8)		N/A^a^		
	Men	39 (37.9)		24 (61.5)		15 (38.5)		N/A		
Age (years), mean (SD)			46.06 (16.9)		45.1 (16.44)		47.4 (17.66)	.51^b^		
**Age (years), n (%)**										
	18-29	20 (19.4)		12 (60.0)		8 (40.0)		>.99		
	30-39	23 (22.3)		15 (65.2)		8 (34.8)		.34		
	40-49	15 (14.6)		7 (46.7)		8 (53.3)		.25		
	50-59	20 (19.4)		13 (65.0)		7 (35.0)		.61		
	≥60	25 (24.3)		13 (52.0)		12 (48.0)		.35		
**Diagnosis, n (%)**										
	All	173 (100.0)		116 (67.1)		57 (32.9)		N/A		
	F1^c^	20 (11.6)		11 (55.0)		9 (45.0)		.32		
	F2^d^	17 (9.8)		6 (35.3)		11 (64.7)		.01		
	F3^e^	53 (30.6)		48 (90.6)		5 (9.4)		<.001		
	F4^f^	36 (20.8)		21 (58.3)		15 (41.7)		.23		
	F6^g^	15 (8.7)		8 (53.3)		7 (46.7)		.25		
	Others^h^	32 (18.5)		22 (68.8)		10 (31.2)		.68		
Number of diagnosis, mean (SD)			1.88 (0.87)		1.95 (0.81)		1.79 (0.94)	.37^b^		

^a^N/A: not applicable.

^b^*P* values were calculated using *t* test, while all other values were calculated based on chi-square test.

^c^F1: Mental and behavioral disorders due to psychoactive substance use.

^d^F2: Schizophrenia, schizotypal, and delusional disorders.

^e^F3: Mood (affective) disorders.

^f^F4: Neurotic, stress-related, and somatoform disorders.

^g^F6: Disorders of adult personality and behavior.

^h^Others: All mental disorders in the International Classification of Diseases-F chapter beyond those previously listed were subsumed under this category.

### Reasons for Acceptance and Rejection of Participation in ORA

The categories and subcategories for the respective reasons provided by the respondents are summarized in Table 2. Subcategories and quotes in the following text are represented in italics.

**Table 2:** Categories and subcategories of stated reasons for acceptance and declination of ORA (N=103).

Categories and subcategories				Values^a^, n (%)
**Reasons for acceptance of ORA^b^**				
	**Wish to engage in treatment**			
		Improved self-understanding and self-knowledge	14 (13.6)	
		Interest in the external perspective provided by clinicians	12 (11.7)	
		Continual contact and exchange	10 (9.7)	
		Incentive for increased engagement in treatment	6 (5.8)	
	**Understanding the treatment process**			
		Reminder of content discussed in therapy sessions	27 (26.2)	
		Ability to track the progress of treatment over time	5 (4.9)	
		Interest in medical translation of own symptoms	3 (2.9)	
	**Critically assessing clinicians**			
		Gaining more transparency into the perspective of health care providers on patients	6 (5.8)	
		Needing to verify the correctness of the notes in order to be able to trust the clinician	10 (9.7)	
		Avoiding and correcting misunderstandings	13 (12.6)	
	**Sharing personal health data with third parties**			
		Improving communication about the illness with significant others	7 (6.8)	
		Ability to share own health data with public institutions	3 (2.9)	
		Having access to own health data	2 (1.9)	
**Reasons for rejection of ORA**				
	**Feeling well supported in face-to-face interactions**			
		Sufficient oral “transmission” of notes	15 (14.6)	
		Desire to address problems and inquiries more effectively in direct conversation	10 (9.7)	
		Adequate satisfaction with in-person appointments	7 (6.8)	
	**Self-uncertainty**			
		Feeling emotionally burdened by reading the notes	16 (15.5)	
		Fear of excessive confrontation with one’s own condition	3 (2.9)	
		Adequate satisfaction with one’s own perspective	7 (6.8)	
	**Uncertainty in the relationship with the clinician**			
		Control weakens trust	2 (1.9)	
		Trust does not require control	19 (18.4)	
		Concern about technical demands for clinicians	1 (1.0)	
	**Concerns about the misuse of health data by third parties**			
		Worries about data security	5 (4.9)	
		Concerns of unwanted control by family members	2 (1.9)	
	**Practical barriers**			
		Difficulties in dealing with technology	13 (12.6)	
		Difficulties in reading and understanding the notes	7 (6.8)	

^a^The number of patients (n, %) who mentioned each theme is indicated in parentheses.

^b^ORA: online record access.

### Reasons for Acceptance

#### Overview

The stated reasons for accepting participation in ORA can be grouped into 4 main categories with a total of 13 subcategories.

#### Wish to Engage in Treatment

The respondents associated their agreement to participate with the wish and motivation to become more actively engaged in their treatment. They hoped that by using the portal, they could gain a better understanding of themselves and their condition, often driven by their interest in the external perspectives of their clinicians.

I am interested in knowing what they actually [think] about me here, because it’s about me, my health. Maybe I can understand everything [about why I’m feeling unwell] better.

Patient 14

In the responses, this interest was often connected to a wish for ongoing communication and interaction.

I find it quite practical because it helps me stay in touch with my doctor and keep track of documentation. This way, I can tell the doctor when I’m not feeling well.

Patient 55

Overall, the participants viewed their participation as an incentive to become more engaged in their treatment.

#### Overview of the Treatment Process

Gaining a comprehensive understanding of the treatment process is another category that emerged from the participants’ responses. While closely related to their willingness to engage in treatment, it primarily focused on the desire to have an overview of the treatment process. Many participants appreciated the ability to read their open notes through ORA, as it served as a reminder of therapy sessions and allowed them to track the chronological progression of their treatment.

I would like to have an overview of how my condition has changed over time, whether things have improved or worsened. Otherwise, you just live in the moment and with the things I tell you in this moment. But having it documented from appointment to appointment, and knowing that things might have gotten better without me realizing it, I would like to have that on paper.

Patient 89

Additionally, embracing ORA was motivated by an interest in understanding how their own symptoms are translated in a medical context. This includes the use of specialized terminology to describe their symptoms and the subsequent treatment recommendations.

I would like to know how you medically process what I tell you during the treatment and what implications it has for the diagnosis. I'm just sharing things from my life, but what does it mean for the illness and what needs to be done now? I would like to see that.

Patient 84

#### Critically Assessing Clinicians

Participants expressed their acceptance of ORA not only as a means to engage more actively in their treatment but also as an opportunity to critically assess the perspectives and approaches of clinicians. Participants valued the chance to gain more transparency about how clinicians view their patients by reading clinical notes and being able to provide feedback via comments.

To find out what therapists think about me behind my back and whether they even notice you in the hospital setting.

Patient 72

Participants expressed concerns that clinicians might not accurately understand or document patients’ individual needs, leading to doubts about whether they can be trusted. This led to a need to verify the correctness of the notes in order to be able to trust their clinician.

I have so much mistrust towards doctors, especially regarding my psychosis and the forced medication, and how things I've said and done have been twisted. I can see it happening with my grandma too, how she's being treated. That's why I just want to see what you actually write down.

Patient 91

From a more constructive perspective, many participants saw the possibility of viewing open notes within ORA as a way of preventing and rectifying misunderstandings that may arise during conversations.

#### Sharing Personal Health Data With Third Parties

This category describes aspects that are less focused on the treatment itself and its documentation, but rather on the use of this documentation with third parties. Participants expressed the hope that sharing the clinical documentation with significant others (eg, family members and friends) and other health care providers would improve the exchange of information about their illness.

For instance, my wife would like to talk to someone about how to handle my condition. That was my first thought, that she could also read what you write. I can't remember and convey everything. This way, she could participate without me burdening her with it.

Patient 27

Furthermore, participants viewed the ability to share their own health data with public institutions such as health insurance companies or the police through ORA as a positive aspect.

I recently had an issue with the health insurance company where they just declared me as healthy. They requested my medical records, but nobody was available at the psychiatric outpatient clinic. I could simply print out my documentation. That would be just great.

Patient 36

Furthermore, the basic opportunity to have access to one’s own health data was also mentioned as a reason for accepting ORA.

### Reasons for Rejection

#### Overview

The reasons for declining participation in ORA can be grouped into 5 categories with 13 subcategories.

#### Feeling Well-Supported in Face-To-Face Interactions

This category includes topics in which participants decline ORA because they already feel adequately cared for through the current mode of contact. For instance, they perceived their in-office appointments as sufficient for their needs.

I am feeling satisfied with conversations. (...) I'm not someone who spends a lot of time on their phone. I prefer being outdoors. (...) If I don't understand something, I can simply ask for clarification. Looking at the notes would only add more information to my already busy mind.

Patient 32

This feeling of being adequately cared for through in-office appointments was repeatedly associated with the desire of the respondents to address problems and inquiries through direct conversation. Moreover, they expressed a preference for discussing their own notes through verbal communication.

We have already discussed it [the topic of today's session]. If there's anything or if I want to know more, I can always ask.

Patient 12

#### Self-Uncertainty

Another central theme in the patients’ statements was the concern or fear of becoming unsettled by reading the notes. While several respondents mentioned being sufficiently satisfied with their own perspective on themselves, there was often an underlying fear of being burdened by reading the notes.

I don't need to read that; I'm already experiencing all this crap myself. I don't need to see it in black and white too.

Patient 78

In this context, some respondents specifically expressed fear of too much confrontation with their own condition, and some of the participants wished to leave that within the scope of the therapeutic conversation and not reactivate it through reading clinical notes.

I wouldn't be up for that. Because, well, I unload all this stuff on you here that makes me sick, and afterwards, I actually feel better. But if I were to read through all that I've told you again, it would really bring me down all over again.

Patient 67

#### Uncertainty in the Relationship to the Clinician

The respondents expressed concerns about not only their own self-uncertainty but also about feeling uncertain toward the health care provider and the therapeutic relationship when it comes to using ORA. Specifically, they highlighted that allowing patients to review their notes could potentially undermine the trust and rapport they have established with their clinicians.

I am a doctor myself and I know that it harms the doctor-patient relationship when patients read what doctors write about them. It is very important to me that I trust you without constantly reviewing what you document.

Patient 92

Contrary to the wish to critically assess the clinician as a justification for the use of ORA (as mentioned above), the respondents emphasized that satisfaction with treatment and a trusting relationship do not require such control.

I trust you that everything is accurate, right? How you write it down. I'm really satisfied with the treatment, I've even had my pension extended, and all the services I need are being provided, so everything you document and how you communicate it to others must be correct, right? Others might want to know sometimes, but I also feel that what I tell you is being understood, so I don't need to read anything extra.

Patient 73

Here, concerns were raised about the potential increase in workload for clinicians, which could potentially strain the therapeutic relationship due to the perceived additional workload.

#### Concerns About the Misuse of Health Data by Third Parties

Some respondents explained their rejection based on concerns about data security, specifically regarding the potentially insecure storage of documentation for instance on mobile devices, which could result in unauthorized access by third parties. Unlike the proponents of ORA, those who expressed opposition to it also raised concerns about unwanted control by care partners through unauthorized access to the patient portal.

I have a curious girlfriend who doesn't necessarily need to read along. (...) It's not for my family members. I would feel too controlled by my partner. She already opens my mail and goes through my bank statements.

Patient 52

#### Practical Barriers

Finally, some respondents mentioned technical and practical challenges as reasons for their rejection. Specifically, participants over the age of 49 years highlighted the difficulty of dealing with the technology required for ORA, such as smartphones, browser apps, and 2-factor authentication. Additionally, some expressed feeling overwhelmed by the comprehension of the notes, as they encountered challenges due to the use of medical terminology and their own difficulties in reading caused by issues with concentration.

I have really bad concentration problems, so that I can't understand anything anymore and can't fully engage in anything, [I] would only understand half of it, especially when reading. Additionally, I don't have internet access, and I don't understand how to set it up.

Patient 97

## Discussion

### Principal Findings

In summary, the reasons provided by the interviewed patients with SMI for their decision to use or not use ORA are diverse. Among those in favor, motivations range from a desire for increased engagement in treatment to critical evaluation of clinicians and using accessible health data for sharing with third parties. In contrast, those who opposed ORA perceived their therapeutic relationship as already well-established and feared that it might be jeopardized by the use of ORA. Finally, practical barriers, mostly related to digital literacy, were cited as reasons for their opposition.

### Acceptance of ORA

The reasons for approval are briefly discussed as they largely align with those found in existing studies, thus providing limited implications for the further development of ORA. Those reasons include the motivation to engage more actively in treatment: by using the portal, patients hope to better understand the content of medical appointments and to obtain a clearer view of themselves, their illness, and the external perspective of their doctors [29]. Although not explicitly thematized in this study, it is reasonable to infer that this motivation also leads to increased adherence to medical treatment. This assumption is supported by another study, which found that patients with SMI when using ORA, reported an improved understanding of their medication prescriptions and described feeling more comfortable and in control throughout the therapeutic process [18,30]. However, this question requires further investigation in a follow-up study.

Moreover, many patients see ORA as a way to obtain a comprehensive overview of their treatment process. This includes accessing open notes as a reminder of therapy sessions, tracking treatment progress, and understanding how their symptoms are documented in a medical context.

Critical evaluation of clinicians is another reason for the acceptance of ORA by patients which is also reported by other studies [31]. The participants in our study reported that by reading the clinical notes, they want to evaluate the transparency and accuracy of clinicians’ perceptions and documentation of their needs. This critical view is also seen as an opportunity to address and correct potential misunderstandings that may occur during the consultation. However, this need for critical monitoring of the practitioners was ultimately linked to the desire to deepen trust in the practitioner and the treatment process. This need or desire to enhance trust is also highlighted in a study by Cromer et al [32].

In addition to patients appreciating gaining access to their health data through ORA, the ability to share personal health information with third parties, such as family, friends, other health care providers and public bodies, is also viewed positively. Again, this finding is consistent with preexisting literature on the perceived benefits of ORA by health care users [33].

### Rejection of ORA

When considering the rejections of ORA among patients with SMI, differences compared to patients from general health settings become apparent. For example, 1 patient acknowledges having significant comprehension difficulties during direct patient-clinician interactions due to concentration problems and suspects that reading clinical notes would exacerbate the issue. This aligns with existing studies that suggest severe symptoms, which persist in daily life and tend to hinder participation in digital health interventions [34]. Furthermore, patients with SMI more often experience intersecting factors such as low educational attainment and language barriers [35], which was also repeatedly stated by study participants with regard to difficulties in dealing with technology and understanding the notes. Low educational attainment and language barriers can subject them to greater stress in patient-clinician interactions when trying to understand health care providers’ explanations [35]. Collectively, these individual limitations can lead to concerns and experiences not being adequately articulated, misunderstood, or possibly forgotten within the limited time available at an appointment. Consequently, this may result in less interest in accessing the notes made by the clinician. On the other hand, these individual limitations could also serve as an argument in favor of ORA: ORA offers the opportunity to enhance understanding of the patient-clinician relationship [36]. It can contribute to mitigating the mentioned disadvantages of inequities by extending the therapeutic interaction beyond physical encounters into the digital space where patients may feel less pressured to conform with the HCP and may also express themselves more easily than in the physical space. However, it is crucial that the clinical notes are written in a language that is relatable to everyday life and nonjudgmental [8,37]. This allows patients to reread and better understand the content discussed during previous appointments in preparation for an upcoming one. Furthermore, a messaging or commenting feature enables patients to ask questions or gather any unresolved concerns. Nonetheless, concern about being emotionally burdened by reading the notes was a common reason for deciding against ORA. Remarkably, these fears correspond with those expectations of HCPs, even though they were rarely confirmed after adopting ORA [3,7,8,12].

These arguments raise the question of whether patients with SMI, who experience daily limitations due to their symptoms, should be informed about the potential benefits of ORA in a more specific or repeated manner, and whether such adapted and improved information could potentially modify the approval rate. On the other hand, it might be that just the opposite is the case and that providers are particularly reluctant to share notes with this population and do in fact not routinely discuss open notes or encourage their clients to read them [38,39]. Unfortunately, this issue did not emerge from our data and further research is needed to clarify this question.

Then there is a group of patients who reject ORA because they already feel well taken care of in face-to-face interactions for various reasons. Studies examining the willingness to adopt digital health services explain the preference for direct patient contact, among other factors, through personality traits [34]. Extraversion, in particular, is considered a predictor of a lower likelihood of engaging with digital health services [40]. Individuals who displayed higher extraversion preferred meeting and connecting with the doctor in person. Some of the statements made by the participants convey a certain persistence in favor of nondigital means of communication (see subcategory “Sufficient oral ‘transmission’ of notes” in Table 2). In line with this, other findings indicate that personality traits associated with resistance to change and openness to new experiences result in a lower adoption of digital health services [41]. Therefore, it would be interesting for further research to explore whether these corresponding personality traits align with the thematic trends found in our study. Beyond these considerations, the attitude of rejecting ORA seems to be explained in particular by the fact of enduring SMI. On the one hand, the hope of indirectly positively influencing one’s own mental health through ORA may be reduced due to the length or severity of the course of illness [34]. On the other hand, in the examined health care system for individuals with SMI, assuming they are in a phase of predominant psychological stability, treatment contacts are rare (approximately 1-2 sessions of 15 minutes each in 3 months). As a result, the opportunities for exchange and the scope of exchangeable content through ORA are limited from the perspective of the surveyed patients [42]. However, it is worth noting that precisely because appointments are short and there are potentially many topics to be discussed (current status of well-being, medications, laboratory results, medication levels, etc), ORA could provide patients with SMI with more space to exchange a wide range of information at a later time and overall enhance the therapeutic contact beyond the physical encounter.

Other participants expressed their rejection of ORA by explaining that the burden of their mental illness in their everyday lives was already substantial, leading them to decline any additional confrontation beyond their appointments at the outpatient clinic. This aspect seems to correspond with the preceding factor, suggesting that these patients are currently unable to dedicate any further (mental) capacity to engage with their chronic mental condition beyond medical appointments.

### Comparison of Acceptance and Rejection of ORA

When comparing the reasons provided by patients for or against the use of ORA, several interesting contrasts become apparent. While some reasons for approval can be interpreted as a desire to deepen the therapeutic relationship, the opposition, in certain cases, stems from the apprehension that this therapeutic alliance may be undermined and jeopardized through the introduction of control (refer to subcategory “Trust does not require control” in Table 2). Conversely, patients who embraced ORA described a high need for control, which motivates their use of ORA. Accordingly, the use of ORA is perceived as an opportunity to critically review the HCP’s perspective and documentation, rather than blindly trusting them (see subcategory “Needing to verify the correctness of the notes in order to be able to trust the clinician” in Table 2). The disclosure of notes, in the optimal scenario, can thus be regarded as a demonstration of trust that allows for a deepening of the therapeutic relationship [42].

Another theme that underlies both the approval and rejection of ORA is the use of health data by third parties. This issue raises concerns about data security and the potential for unwanted control by family members when sharing information with significant others, health care providers, and public institutions. It is important to note that privacy and trustworthiness are among the most common reservations regarding ORA and digital (mental) health interventions in general, given the sensitive and potentially stigmatizing nature of the content involved [16,34,43]. A recent study conducted in Sweden provides evidence that these reservations are valid, as patients with mental illness experience significantly more attempts by unauthorized individuals to access their mental health records compared to patients in general health settings [44]. In our study, 1 patient expressed the misconception that health data are directly stored on their mobile phone. This misunderstanding highlights a knowledge gap where patients may not be aware that the data are actually securely stored remotely using 2-factor authentication, thereby aiming to prevent unauthorized access through the phone. However, the concerns expressed in our study once again emphasize the importance of data protection in the implementation of digital health platforms and the need for sufficient patient and provider education on this matter.

### Differences Between Patient Groups

Generally, the proportion of patients willing to use ORA is approximately 60%, which is consistent with findings from previous studies [19,43,45]. However, the actual usage rate of ORA among patients is expected to be even lower [18]. The 2 groups, those in favor and those against ORA show little difference in terms of age, gender, distribution of diagnoses, and comorbidity, except for psychotic and depressive disorders. The higher levels of agreement and motivation among patients with depression align with findings from other studies [43,45], possibly due to a higher prevalence of socially desirable behavior in this patient group. The low approval rate among patients with schizophrenia is somewhat surprising compared to the existing literature. According to previous studies, patients with psychosis are generally very well able to use web-based interventions, exhibit positive attitudes toward them, and use the web-based more frequently to build their social networks compared to the general population [46-48]. The rejection of ORA in our study population could be attributed to reduced digital literacy, functional impairments caused by psychotic symptoms, as well as an approach to the illness characterized by internalized stigma and social withdrawal [49]. This social withdrawal has also been described as a protective mechanism against overly social and open interactions [50].

### Strengths and Limitations

This is the first study that examines the reasons for the acceptance and rejection of ORA among patients with SMI in the German health care system. The investigation of these factors is crucial for advancing the implementation of ORA in the German-speaking region and can only be meaningful through a comparison with international research findings. Moreover, the study contributes to filling the research gap regarding the perspectives of individuals with SMI toward ORA.

One limitation of the study is that while a variety of reasons for rejecting ORA became apparent in the qualitative survey, raising further questions about factors such as digital literacy or the respondents’ social behavior, these factors were not explored in the quantitative survey. Similarly, comprehensive sociodemographic information such as educational level, socioeconomic status, or duration of mental illness was unfortunately not available in the data corpus. A follow-up study may be useful to further validate the qualitative data and to analyze in-depth the role of other patient characteristics that contribute to the decision for or against ORA.

Another limitation of the study is that it did not present the proportion of patients who actually used ORA after having consented to do so. Since this study is based on baseline data from the PEPPPSY study [21], the analysis of usage patterns and effects of ORA is yet to be conducted. Another issue at first glance is the dichotomization of the results (see Table 2). The question arises as to how any positive attitudes of patients who reject ORA (and vice versa) were taken into account. For instance, patients who opt for ORA may still hold concerns regarding data privacy. However, this limitation was addressed by incorporating any sub-aspects of patient statements that are in opposition to their decision for or against ORA in the qualitative analysis of the data. This means that all patient statements and attitudes toward ORA were accounted for in our qualitative analysis independently from the patients’ decisions for or against ORA.

### Future Research

In addition to the research gaps identified above, further research is needed to address the unique needs of individuals with SMI in order to effectively facilitate maintained engagement with ORA. First, the extent to which patient characteristics and, in particular, psychiatric functional impairments, as well as concepts such as internalized stigma and social withdrawal, influence acceptance of ORA should be investigated further. Possible influences of personality traits such as extraversion or resistance to change on willingness to use ORA should also be considered.

Second, it should be investigated to a larger extent, whether the fear of possible adverse effects from reading the findings and clinical notes made available via ORA is confirmed in practice. Although studies to date tend to suggest otherwise, patients’ concerns should be taken seriously. In this context, research should be conducted on how to formulate clinical notes in a way that is both understandable and empathetic to patients without overburdening the available resources of practitioners. In this respect, there are preliminary indications of promising use of generative language models [51].

Third, there is no evidence on what cues, explanations, or motivations patients with SMI need from the medical team, and especially from their clinicians, to want to use ORA more. In this context, it should be investigated whether improved patient information about the benefits of ORA increases adoption rates. At the same time, there seems to be a need to explore what skills HCPs need to acquire in order to formulate clinical notes in a way that is understood by patients and adds value, which involves adapting their communication style to align with patients’ familiar vocabulary rather than relying solely on technical medical terminology. Finally, actual rates of ORA use among patients with SMI compared to adoption rates and reasons for potential discrepancies should be explored.

### Conclusions

In general, patients with affective disorders (ICD 10, F3) showed high interest in ORA, whereas patients with schizophrenia, schizotypal and delusional disorders (ICD 10, F2) were less interested. It was mainly female patients of younger (18-39 years) and middle (50-59 years) age who agreed to receive ORA. Acceptance of ORA by patients with SMI stems primarily from a desire to be more actively involved in their care, to have a comprehensive view of their treatment process, and to evaluate the accuracy of physicians’ perception and documentation of their needs. This critical perspective is also seen as an opportunity to address and correct any misunderstandings that may have occurred during the consultation. The value placed on access to personal health information, combined with the ability to share that information with third parties, underscores the patients’ positive attitudes toward ORA.

Rejection of ORA by patients with SMI is primarily motivated by a sense of already being well supported by face-to-face interactions, as well as concerns rooted in their own insecurities. These range from fear of being unsettled by reading clinical notes to avoidance of excessive confrontation with one’s condition outside of the therapeutic conversations. Patients worry that the transparency created by ORA could undermine trust in their health care providers, especially given the additional workload for clinicians. Finally, data security risks and practical barriers such as lack of digital literacy and incomprehensible medical jargon contributed to the decision not to use ORA.

## Appendix

Multimedia Appendix 1 Interview guidelines.

Multimedia Appendix 2 COREQ (Consolidated Criteria for Reporting Qualitative Research) checklist.

## Abbreviations

COREQ: Consolidated Criteria for Reporting Qualitative research
HCP: health care professional
ICD: International Classification of Diseases
MHB: Brandenburg Medical School
NTNU: Norwegian University of Science and Technology
ORA: online record access
PEPPPSY: Piloting and Evaluation of a Participatory Patient-Accessible Electronic Health Record in Psychiatry and Somatics
SMI: severe mental illness
