# Supplementary material for: Reasons for Acceptance or Rejection of Online Record Access Among Patients Affected by a Severe Mental Illness: Mixed Methods Study
Source: JMIR Ment Health. 2024 Feb 5;11:e51126. doi: 10.2196/51126 (PMC10877495; doi:10.2196/51126)
Supplement: Multimedia Appendix 1 [file mental_v11i1e51126_app1.docx]

**Interview guide**

You have decided in favor of/against gaining access to your practitioners' progress notes via a secure online portal as part of a study. We would like to better understand your decision and your motivations behind it. To that end, we would like to ask you a few follow-up questions below:

I would like to know why you would like to use this / don't need to?

Could you please explain in more detail what motivated you to (not) want to view your practitioners' clinical notes online?

- To what extent does your decision for or against ORA have something to do with certain expectations or also fears regarding your further course of treatment?
- To what extent does your decision for or against ORA have something to do with certain expectations or fears regarding the relationship with your therapist?
- To what extent does your decision for or against ORA have something to do with certain expectations or fears regarding your self-perception?
- What other reasons played a role for you?

If you have decided in favor of ORA:

- Can you think of possible scenarios for how you will use ORA? Based on what particular features of the electronic record did you choose to access?

If you decided against ORA:

- Were there any concerns or reservations you have about online record access that may have influenced your decision?
- Might you have needed more information? If yes, what was it?
